# Chronology of permanent teeth mineralization in Brazilian individuals: age estimation tables

**DOI:** 10.1186/s12903-023-02837-y

**Published:** 2023-03-22

**Authors:** Barbara Kuhnen, Clemente Maia da Silva Fernandes, Franciéllen de Barros, José Scarso Filho, Marcelo Gonçalves, Mônica da Costa Serra

**Affiliations:** 1 Department of Community Dentistry, School of Dentistry, Araraquara, São Paulo State University – UNESP, Rua Humaitá 1680, Araraquara, SP 14.801-903 Brazil; 2 Department of Diagnosis and Surgery, School of Dentistry, Araraquara, São Paulo State University – UNESP, Rua Humaitá 1680, Araraquara, SP 14.801-903 Brazil

**Keywords:** Forensic sciences, Forensic dentistry, Forensic anthropology, Age determination by teeth

## Abstract

**Background:**

Age is important in forming the uniqueness of individuals. When chronological age is not available, age estimation is required, particularly in court cases. The mineralization chronology of permanent teeth is a valuable tool for age estimation of subadults. This study aimed to evaluate the mineralization stages of permanent teeth of Brazilian subjects from imaging exams, using the classification by Moorrees et al. modified by the authors, to verify the existence of correlation between the chronology of mineralization stages and sex and to prepare numerical tables of the chronology of dental mineralization stages for Brazilian individuals.

**Methods:**

Digital panoramic radiographs of 1100 living Brazilian individuals of both sexes, aged between 2 and 25 years, born between 1990 and 2018, from the image bank of a Dental Radiographs and Documentations clinic located in the city of Araraquara, SP, Brazil. The images were evaluated according to the level of crown and root development and classified according to the stages proposed by Moorrees et al. (Am J Phys Anthropol 21: 205–213, 1963) adapted by the authors. All analyses were performed in the R software. Descriptive and exploratory analyses were performed on all data. For intra- and inter-examiner analyses, the rate of agreement and Kappa statistics at a 95% confidence interval were used. Kappa was interpreted according to Landis and Koch.

**Results:**

Only upper and lower canines showed significant differences between the sexes (p < 0.05), with higher average ages for men. The findings were presented in tables, as well as age estimates with 95% confidence intervals for each mineralization stage and each tooth.

**Conclusion:**

In the present study, we evaluated the mineralization stages of permanent teeth of Brazilian subjects from digital panoramic radiographs and found no correlation between the chronology of mineralization stages and sex, except for canines. From the obtained results, numerical tables of the chronology of dental mineralization stages were prepared.

## Background

Age is important in forming the uniqueness of individuals. When the chronological age cannot be determined, age estimation is required, particularly in court cases. Expert age estimation investigations have been increasingly necessary for living individuals besides the traditional performance to compose, along with data on sex, ancestry, and stature estimates, the biological profile for human identification.

The increase in migratory movements in the early 2000s triggered a growing demand for age estimation in living individuals [1], becoming crucial in countries receiving many immigrants, as several people enter countries undocumented [2]. Estimating age may also be needed to assist law enforcement authorities in cases of human identification, age estimation at death, search for unknown victims, and determination of eligibility for social benefits [3].

Several studies have demonstrated the reliability of using human teeth to estimate chronological age because they are less likely to undergo external events than other body structures. The scientific literature highlights dental analysis for age estimation, especially for young individuals [2], as the dental techniques used in this population are more accurate than other methods [4].

In subadults, dental age is usually estimated by comparing the pattern of dental development of the individual in question with the data researched in samples of subjects with known ages. Most methods developed for age estimation are based on the comparison between the dental development observed with the analysis of intraoral and extraoral radiographs and standardized tables from studies with different populations [5, 6].

Some authors such as Nolla [7], Moorrees et al. [8], Demirjian et al. [9], and Willems et al. [10] studied the mineralization of permanent teeth and proposed methods to estimate the age of subadults. The authors established different mineralization stages. Unfortunately, there are no numerical tables with average ages for the different mineralization stages proposed.

Nolla [7] proposed 10 stages. A maturity score is obtained by adding the stages of teeth analyzed, and the estimated age is based on the score, using the conversion tables proposed by the author for female and male subjects [6].

Moorrees et al. [8] proposed 13 mineralization stages for single-rooted teeth and 14 stages for molars. These authors presented graphic representations, for both sexes, of the mineralization stages of permanent teeth. The numerical parameters were not available, which limited or hindered their application [11]. However, other authors used the stages established to develop numerical tables for tooth formation stages. These authors include Phillips and van Wyk Kotze [12], who developed tables with numerical values of average ages for the South African population; and Karkhanis et al. [13], who proposed values for the Australian population.

Gleiser and Hunt [14], studying the formation of first mandibular molars, proposed 15 mineralization stages; and Haavikko [15] adapted the stages proposed by the authors aforementioned and reduced them to 12 stages, presenting numerical values for the mineralization stages of permanent teeth.

Demirjian et al. [9] established eight dental mineralization stages and proposed tables in which the stages of each tooth analyzed are converted into a specific score. A maturity score is obtained by adding the scores of the teeth, and conversion tables transform them into the estimated dental age. Willems et al. [10] adapted the Demirjian method using the same eight stages, but each one receives a new score. The resulting sum of the scores of teeth of a hemiarch directly provides the estimated age [6].

For the Brazilian population, researchers Nicodemo, Moraes, and Médici Filho, in 1974, created a table for the chronology of permanent teeth mineralization. At that time, the authors had observed that foreign data in the literature were not compatible with the Brazilian sample, and there was a need for national standards of dental age assessments. Each researcher worked on some groups of dental elements in isolation. Nicodemo studied the mineralization of third molars with periapical and extraoral radiographs, Moraes studied first molars and incisors, and Médici Filho studied second molars, canines, and premolars [16]. These authors used eight dental mineralization stages based on the 10 stages proposed by Nolla [7]. The findings of their studies were gathered in a single table of permanent teeth mineralization, known as N.M.M., with numerical data on maximum and minimum age in months for dental age characterization [17].

Nevertheless, studies have shown low accuracy rates for age estimation in Brazilian individuals using the N.M.M. table [16, 18, 19] and methods proposed by international authors [20].

Ubelaker and Parra [21], Santoro et al. [22], Karkhanis et al. [13] and Koshy and Tandon [5] emphasize that age estimation is highly accurate when methods and equations are used for a specific population. Thus, method reliability increases when applied to different population groups [2, 5, 13].

Differences in ethnic and geographic origins show small variations in dental development, thus standards and formulas for age estimation should be specific to populations or regions [5, 13, 21–24]. Further assessments in modern Brazilian subpopulations are required for a more reliable application of age estimation methods.

Thus, studies verifying the current chronologies of the stages proposed by different authors are indicated. For instance, a recent study by Šešelj et al. [25] proposed a new chronology of dental development using the stages proposed by Moorrees et al. [8].

Up-to-date information is required in the search for more reliable age estimation expertise, as well as research studies verifying the current dental mineralization stages for the Brazilian population.

The present study aimed to evaluate the mineralization stages of permanent teeth of Brazilian subjects from imaging exams, using the classification by Moorrees et al. modified by the authors, to verify the existence of correlation between the chronology of mineralization stages and sex and to prepare numerical tables of the chronology of dental mineralization stages for Brazilian individuals.

## Methods

### Sample

The sample of this study consisted of digital panoramic radiographs of 1100 living Brazilian individuals of both sexes, aged between 2 and 25 years (35–307 months), born between 1990 and 2018, from the image bank of a Dental Radiographs and Documentations clinic located in the city of Araraquara, SP, Brazil.

The images presented all permanent teeth of upper and lower hemiarches (erupted or not). For standardization purposes, the left hemiarches were evaluated. According to Nolla [7], the values for one side are representative of the development of the teeth of the maxilla and the mandible.

Images that did not provide good visualization of the teeth or with missing teeth in both hemiarches were excluded. A total of 1004 radiographs were included—502 from men and 502 from women. Table 1 shows the age distribution of the sample.

**Table 1:** Sample age distribution

Age (in years)	n
2	4
3	13
4	39
5	62
6	63
7	70
8	44
9	37
10	33
11	28
12	36
13	46
14	36
15	47
16	39
17	47
18	44
19	48
20	53
21	44
22	47
23	50
24	52
25	22
Total	1004

This study was approved by the Human Research Ethics Committee of the School of Dentistry of Araraquara – UNESP (CAAE n. 47710721.0.0000.5416).

### Analysis of dental mineralization stages

The study analyzed the mineralization stages of permanent teeth in the upper left (21, 22, 23, 24, 25, 26, 27, and 28) and lower left (31, 32, 33, 34, 35, 36, 37, and 38) hemiarches. In the case of a missing tooth on the quadrant side, the corresponding tooth on the right quadrant was analyzed.

**Fig. 1:**
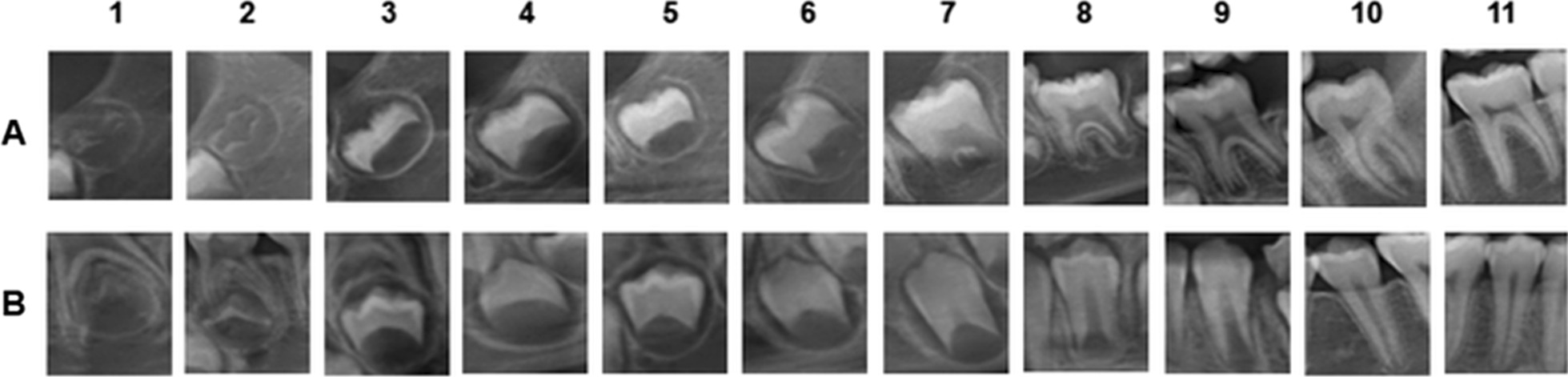
Classification of mineralization stages of permanent teeth. A. Molars. B. Single-rooted teeth. 1. CI 2. COC 3. CR1/2 4. CR3/4 5. CRC 6. RI 7. R1/4 8. R1/2 9. R3/4 10. RC 11. AC

The radiographic images of permanent teeth were evaluated according to the level of crown and root development and classified according to the stages proposed by Moorrees et al. [8] adapted by the authors, using 11 different stages (Fig. 1). Moorrees et al. [8] proposed 14 mineralization stages, which were reduced to 11 stages, in order to simplify and to facilitate the process. The stage “coalescence of cusps” was included in “initial cusp formation”; the stage “initial cleft formation” was considered as “initial root formation”, and the stage “apex ½ closed” was included in the “complete apex” stage.

1. CI: Initial cusp formation
2. COC: Complete Cusp Contour
3. CR1/2: ½ crown
4. CR3/4: ¾ crown
5. CRC: Full Crown
6. RI: Initial Root Formation
7. R1/4: ¼ root
8. R1/2: ½ root
9. R3/4: ¾ root
10. RC: Complete root
11. AC: Complete apex

The digital panoramic radiographs were coded, so the examiners did not know the chronological age of each individual.

For the analysis of reproducibility, two trained and calibrated examiners experienced in interpreting radiographic images evaluated 30 radiographs (which were part of the total study sample of 1004 panoramics), randomly chosen, at intervals of at least seven days. The panoramic radiographs were analyzed by the previously calibrated examiners, who were blind to the chronological age or sex of the subjects.

### Statistical analysis

All analyses were performed in the R software [26]. Descriptive and exploratory analyses were performed on all data. For intra- and inter-examiner analyses, the rate of agreement and Kappa statistics at a 95% confidence interval were used. Kappa was interpreted according to Landis and Koch [27]. The sample distribution in the different stages was described with absolute and relative frequencies. Average ages and standard deviations were calculated, in months, for each stage and each tooth. For stages with at least 10 individuals of each sex, the sexes were compared according to age. Hence, Welch's t-test with Bonferroni correction was used. Also in these cases with at least 10 individuals, 95% confidence intervals were calculated for the ages at each stage. The distribution of stages according to sex for teeth 28 and 38 was analyzed with Fisher's exact test. All analyses considered a 5% significance level.

## Results

Intra-examiner agreement in the classification of dental mineralization stages ranged from 87.0 to 100.0%, with Kappa ranging from 0.92 to 1.00, which is an almost perfect agreement according to Landis and Koch [27]. Inter-rater agreement ranged from 92.0 to 100.0%, with Kappa ranging from 0.92 to 1.00, which is also an almost perfect agreement.

**Fig. 2:**
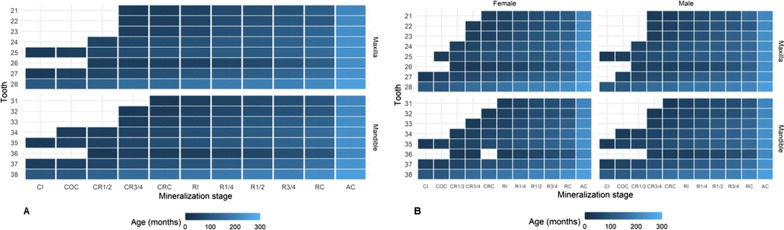
**A** Schematic representation of age variation regarding the dental mineralization stage in Brazilian individuals of both sexes, aged 2–25 years (35–307 months). **B** Schematic representation of age variation regarding the dental mineralization stage and sex in Brazilian individuals

Figure 2A shows the age distribution (the darker, the younger) for each mineralization stage; Fig. 2B presents these findings for each sex separately.

Table 2 shows the sampling distribution for each tooth and sex according to the mineralization stage. Table 3 presents the average age (in months), standard deviation and 95% confidence intervals of Brazilian individuals aged 2 to 25 years (35 to 307 months) for each mineralization stage of each tooth, according to sex and for the total sample.

**Table 2:** Frequency distribution of the sample of Brazilian individuals aged between 2 and 25 years (35 and 307 months) according to the mineralization stage of each tooth, for males and females

Tooth	Sex	Mineralization stage										
		CI	COC	CR1/2	CR3/4	CRC	RI	R1/4	R1/2	R3/4	RC	AC
21	F	0 (0.0%)	0 (0.0%)	0 (0.0%)	0 (0.0%)	2 (0.4%)	36 (7.3%)	27 (5.5%)	47 (9.5%)	16 (3.2%)	11 (2.2%)	356 (71.9%)
	M	0 (0.0%)	0 (0.0%)	0 (0.0%)	1 (0.2%)	2 (0.4%)	33 (6.7%)	33 (6.7%)	44 (9.0%)	24 (4.9%)	6 (1.2%)	346 (70.8%)
22	F	0 (0.0%)	0 (0.0%)	0 (0.0%)	1 (0.2%)	10 (2.1%)	41 (8.5%)	43 (8.9%)	26 (5.4%)	14 (2.9%)	11 (2.3%)	339 (69.9%)
	M	0 (0.0%)	0 (0.0%)	0 (0.0%)	2 (0.4%)	13 (2.7%)	42 (8.7%)	46 (9.5%)	23 (4.8%)	12 (2.5%)	14 (2.9%)	331 (68.5%)
23	F	0 (0.0%)	0 (0.0%)	0 (0.0%)	11 (2.2%)	22 (4.4%)	32 (6.5%)	59 (11.9%)	17 (3.4%)	30 (6.1%)	14 (2.8%)	310 (62.6%)
	M	0 (0.0%)	0 (0.0%)	0 (0.0%)	9 (1.8%)	36 (7.2%)	40 (8.0%)	55 (11.1%)	21 (4.2%)	28 (5.6%)	13 (2.6%)	295 (59.4%)
24	F	0 (0.0%)	0 (0.0%)	10 (2.2%)	63 (14.1%)	27 (6.0%)	25 (5.6%)	32 (7.2%)	17 (3.8%)	5 (1.1%)	5 (1.1%)	263 (58.8%)
	M	0 (0.0%)	0 (0.0%)	16 (3.4%)	78 (16.8%)	32 (6.9%)	20 (4.3%)	27 (5.8%)	6 (1.3%)	9 (1.9%)	6 (1.3%)	271 (58.3%)
25	F	0 (0.0%)	1 (0.2%)	62 (13.6%)	30 (6.6%)	18 (4.0%)	17 (3.7%)	24 (5.3%)	14 (3.1%)	9 (2.0%)	8 (1.8%)	272 (59.8%)
	M	1 (0.2%)	2 (0.4%)	81 (17.6%)	29 (6.3%)	13 (2.8%)	14 (3.0%)	23 (5.0%)	6 (1.3%)	11 (2.4%)	5 (1.1%)	276 (59.9%)
26	F	0 (0.0%)	0 (0.0%)	3 (0.6%)	3 (0.6%)	4 (0.8%)	10 (2.0%)	24 (4.9%)	42 (8.6%)	60 (12.2%)	38 (7.7%)	307 (62.5%)
	M	0 (0.0%)	0 (0.0%)	3 (0.6%)	2 (0.4%)	6 (1.2%)	6 (1.2%)	27 (5.5%)	47 (9.6%)	69 (14.1%)	27 (5.5%)	302 (61.8%)
27	F	1 (0.2%)	10 (2.0%)	45 (9.1%)	18 (3.6%)	37 (7.5%)	38 (7.7%)	16 (3.2%)	12 (2.4%)	9 (1.8%)	36 (7.3%)	273 (55.2%)
	M	0 (0.0%)	12 (2.4%)	51 (10.3%)	24 (4.9%)	28 (5.7%)	43 (8.7%)	17 (3.4%)	7 (1.4%)	9 (1.8%)	24 (4.9%)	278 (56.4%)
28	F	11 (3.8%)	9 (3.1%)	20 (6.8%)	17 (5.8%)	19 (6.5%)	26 (8.9%)	22 (7.5%)	13 (4.4%)	14 (4.8%)	27 (9.2%)	115 (39.2%)
	M	9 (3.1%)	7 (2.4%)	20 (6.9%)	14 (4.8%)	15 (5.2%)	25 (8.7%)	14 (4.8%)	15 (5.2%)	20 (6.9%)	19 (6.6%)	131 (45.3%)
31	F	0 (0.0%)	0 (0.0%)	0 (0.0%)	0 (0.0%)	0 (0.0%)	8 (1.7%)	33 (6.8%)	34 (7.0%)	29 (6.0%)	4 (0.8%)	376 (77.7%)
	M	0 (0.0%)	0 (0.0%)	0 (0.0%)	0 (0.0%)	1 (0.2%)	12 (2.5%)	32 (6.6%)	56 (11.5%)	18 (3.7%)	6 (1.2%)	363 (74.4%)
32	F	0 (0.0%)	0 (0.0%)	0 (0.0%)	0 (0.0%)	5 (1.0%)	16 (3.3%)	42 (8.7%)	35 (7.2%)	10 (2.1%)	17 (3.5%)	359 (74.2%)
	M	0 (0.0%)	0 (0.0%)	0 (0.0%)	1 (0.2%)	5 (1.0%)	18 (3.7%)	48 (9.9%)	39 (8.0%)	7 (1.4%)	8 (1.6%)	359 (74.0%)
33	F	0 (0.0%)	0 (0.0%)	0 (0.0%)	7 (1.4%)	11 (2.2%)	36 (7.3%)	63 (12.8%)	20 (4.1%)	28 (5.7%)	13 (2.6%)	315 (63.9%)
	M	0 (0.0%)	0 (0.0%)	0 (0.0%)	6 (1.2%)	18 (3.6%)	55 (11.0%)	58 (11.6%)	25 (5.0%)	26 (5.2%)	13 (2.6%)	298 (59.7%)
34	F	0 (0.0%)	0 (0.0%)	10 (2.0%)	19 (3.8%)	28 (5.7%)	44 (8.9%)	40 (8.1%)	21 (4.2%)	20 (4.0%)	9 (1.8%)	304 (61.4%)
	M	0 (0.0%)	1 (0.2%)	7 (1.4%)	29 (5.8%)	38 (7.6%)	48 (9.6%)	40 (8.0%)	14 (2.8%)	10 (2.0%)	10 (2.0%)	302 (60.5%)
35	F	2 (0.4%)	2 (0.4%)	19 (3.9%)	32 (6.5%)	35 (7.1%)	30 (6.1%)	35 (7.1%)	10 (2.0%)	18 (3.7%)	20 (4.1%)	287 (58.6%)
	M	2 (0.4%)	6 (1.2%)	22 (4.4%)	38 (7.7%)	44 (8.9%)	31 (6.2%)	27 (5.4%)	8 (1.6%)	12 (2.4%)	17 (3.4%)	289 (58.3%)
36	F	0 (0.0%)	0 (0.0%)	2 (0.4%)	4 (0.8%)	0 (0.0%)	2 (0.4%)	28 (5.6%)	23 (4.6%)	50 (10.0%)	33 (6.6%)	356 (71.5%)
	M	0 (0.0%)	0 (0.0%)	0 (0.0%)	4 (0.8%)	2 (0.4%)	4 (0.8%)	21 (4.3%)	35 (7.1%)	58 (11.7%)	28 (5.7%)	342 (69.2%)
37	F	3 (0.6%)	6 (1.2%)	51 (10.3%)	32 (6.5%)	23 (4.7%)	25 (5.1%)	30 (6.1%)	5 (1.0%)	18 (3.6%)	35 (7.1%)	266 (53.8%)
	M	4 (0.8%)	7 (1.4%)	59 (11.8%)	41 (8.2%)	25 (5.0%)	11 (2.2%)	30 (6.0%)	5 (1.0%)	14 (2.8%)	38 (7.6%)	264 (53.0%)
38	F	13 (4.3%)	13 (4.3%)	20 (6.6%)	25 (8.3%)	15 (5.0%)	15 (5.0%)	14 (4.7%)	24 (8.0%)	13 (4.3%)	37 (12.3%)	112 (37.2%)
	M	14 (4.4%)	7 (2.2%)	25 (7.9%)	17 (5.4%)	16 (5.1%)	17 (5.4%)	13 (4.1%)	24 (7.6%)	18 (5.7%)	22 (7.0%)	142 (45.1%)

*F* Female; *M* Male

**Table 3:** Average age (in months), standard deviation and 95% confidence intervals of Brazilian individuals aged 2–25 years (35–307 months) for each mineralization stage of each tooth, according to sex and for the total sample

Tooth	Sex	CI			COC			CR1/2			CR3/4			CRC		
		A	SD	CI	A	SD	CI	A	SD	CI	A	SD	CI	A	SD	CI
21	F	–	–	–	–	–	–	–	–	–	–	–	–	49.15	16.95	–103.12–201.41
21	M	–	–	–	–	–	–	–	–	–	40.97	–	–	43.88	4.50	3.45–84.32
21	Total	–	–	–	–	–	–	–	–	–	40.97	–	–	46.51	10.57	29.69–63.33
21	p-value	–			–			–			–			–		
22	F	–	–	–	–	–	–	–	–	–	42.58	–	–	49.86	10.98	42.01–57.72
22	M	–	–	–	–	–	–	–	–	–	48.74	1.22	37.75–59.72	52.77	11.50	45.82–59.72
22	Total	–	–	–	–	–	–	–	–	–	46.68	3.66	37.60–55.77	51.50	11.12	46.70–56.31
22	p-value	–			–			–			–			1.0000		
23	F	–	–	–	–	–	–	–	–	–	47.56	10.64	40.41–54.71	56.02	9.48	51.82–60.22
23	M	–	–	–	–	–	–	–	–	–	46.86	8.88	40.03–53.68	62.97	9.29	59.82–66.11
23	Total	–	–	–	–	–	–	–	–	–	47.24	9.64	42.73–51.76	60.33	9.88	57.73–62.93
23	p-value	–			–			–			–			0.8367		
24	F	–	–	–	–	–	–	52.27	12.29	43.48–61.06	65.86	12.96	62.59–69.12	81.06	10.95	76.72–85.39
24	M	–	–	–	–	–	–	59.17	16.06	50.61–67.72	72.33	13.79	69.22–75.44	88.77	14.01	83.72–93.83
24	Total	–	–	–	–	–	–	56.51	14.86	50.51–62.51	69.43	13.76	67.14–71.73	85.24	13.18	81.81–88.68
24	p-value	–			–			1.0000			0.4709			1.0000		
25	F	–	–	–	37.16	–	–	65.18	12.11	62.10–68.25	77.34	13.78	72.20–82.49	96.57	16.34	88.45–104.70
25	M	35.35	–	–	48.72	10.96	–49.76–147.19	72.39	13.64	69.37–75.40	85.99	15.08	80.26–91.73	95.44	13.90	87.04–103.84
25	Total	35.35	–	–	44.86	10.23	19.46–70.27	69.26	13.44	67.04–71.48	81.60	14.96	77.70–85.49	96.10	15.13	90.55–101.65
25	p-value	–			–			0.1092			1.0000			1.0000		
26	F	–	–	–	–	–	–	37.83	4.12	27.60–48.06	38.51	3.65	29.45–47.58	49.06	2.54	45.03–53.10
26	M	–	–	–	–	–	–	52.43	25.09	–9.89–114.75	58.37	25.80	–173.46–290.21	53.00	5.10	47.65–58.35
26	Total	–	–	–	–	–	–	45.13	17.96	26.28–63.98	46.46	17.07	25.26–67.66	51.43	4.55	48.17–54.68
26	p-value	–			–			–			–			–		
27	F	35.73	–	–	55.94	5.24	52.19–59.68	63.90	9.91	60.93–66.88	72.18	11.49	66.47–77.89	90.29	11.13	86.58–94.00
27	M	–	–	–	53.47	4.47	50.63–56.31	67.65	10.73	64.63–70.66	80.64	7.48	77.48–83.80	87.94	9.75	84.15–91.72
27	Total	35.73	–	–	54.59	4.88	52.43–56.76	65.89	10.47	63.77–68.01	77.01	10.20	73.83–80.19	89.28	10.55	86.66–91.89
27	p-value	–			1.0000			1.0000			1.0000			1.0000		
28	F	111.96	11.37	104.32–119.60	107.63	23.74	89.38–125.88	131.51	22.08	121.18–141.85	158.51	18.42	149.05–167.98	151.42	22.92	140.38–162.47
28	M	110.23	14.99	98.71–121.75	114.81	10.31	105.27–124.35	133.13	17.00	125.17–141.08	156.24	16.12	146.93–165.54	151.62	20.47	140.29–162.96
28	Total	111.18	12.79	105.20–117.16	110.77	18.89	100.71–120.83	132.32	19.47	126.09–138.55	157.49	17.17	151.19–163.78	151.51	21.55	143.99–159.03
28	p-value	–			–			1.0000			1.0000			1.0000		
31	F	–	–	–	–	–	–	–	–	–	–	–	–	–	–	–
31	M	–	–	–	–	–	–	–	–	–	–	–	–	35.35	–	–
31	Total	–	–	–	–	–	–	–	–	–	–	–	–	35.35	–	–
31	p-value	–			–			–			–			–		
32	F	–	–	–	–	–	–	–	–	–	–	–	–	37.28	3.05	33.49–41.07
32	M	–	–	–	–	–	–	–	–	–	35.35	–	–	44.64	4.03	39.63–49.64
32	Total	–	–	–	–	–	–	–	–	–	35.35	–	–	40.96	5.14	37.28–44.63
32	p-value	–			–			–			–			–		
33	F	–	–	–	–	–	–	–	–	–	41.40	7.49	34.47–48.32	54.45	8.45	48.78–60.13
33	M	–	–	–	–	–	–	–	–	–	49.31	10.64	38.15–60.48	57.81	9.52	53.07–62.54
33	Total	–	–	–	–	–	–	–	–	–	45.05	9.60	39.25–50.85	56.53	9.12	53.06–60.00
33	p-value	–			–			–			–			1.0000		
34	F	–	–	–	–	–	–	43.69	7.21	38.53–48.85	54.26	6.18	51.29–57.24	65.49	7.30	62.65–68.32
34	M	–	–	–	40.97	–	–	50.85	10.17	41.44–60.25	60.78	10.50	56.79–64.77	72.98	10.86	69.41–76.55
34	Total	–	–	–	40.97	–	–	46.64	9.01	42.00–51.27	58.20	9.52	55.44–60.97	69.80	10.16	67.30–72.30
34	p-value	–			–			–			0.8742			0.1374		
35	F	36.20	1.36	23.95–48.45	53.42	7.66	–15.44–122.28	53.67	7.89	49.86–57.47	64.43	8.62	61.33–67.54	82.22	12.66	77.87–86.57
35	M	40.83	0.19	39.14–42.53	56.41	14.58	41.11–71.71	60.63	11.83	55.38–65.87	69.58	10.76	66.04–73.12	87.05	13.71	82.88–91.22
35	Total	38.52	2.79	34.07–42.96	55.66	12.73	45.02–66.31	57.40	10.67	54.03–60.77	67.23	10.11	64.82–69.64	84.91	13.39	81.91–87.91
35	p-value	–			–			1.0000 1.0000						1.0000		
36	F	–	–	–	–	–	–	35.71	0.04	35.35–36.06	40.54	5.42	31.92–49.16	–	–	–
36	M	–	–	–	–	–	–	–	–	–	54.83	32.29	3.45–106.21	44.02	4.31	5.28–82.76
36	Total	–	–	–	–	–	–	35.71	0.04	35.35–36.06	47.69	22.75	28.66–66.71	44.02	4.31	5.28–82.76
36	p-value	–			–			–			–			–		
37	F	48.02	12.72	16.43–79.61	50.92	4.74	45.95–55.89	63.04	9.36	60.41–65.68	82.71	11.36	78.61–86.81	92.43	15.13	85.88–98.97
37	M	44.54	4.65	37.13–51.95	58.62	12.88	46.71–70.53	67.71	10.21	65.05–70.37	89.27	12.36	85.37–93.17	94.57	15.08	88.35–100.79
37	Total	46.03	8.26	38.39–53.67	55.06	10.40	48.78–61.35	65.55	10.06	63.65–67.45	86.40	12.29	83.53–89.26	93.54	14.98	89.19–97.89
37	p-value	–			–			1.0000			1.0000			1.0000		
38	F	105.15	14.80	96.21–114.10	106.37	19.96	94.31–118.43	138.40	18.40	129.79–147.02	153.40	17.35	146.23–160.56	163.14	20.95	151.54–174.74
38	M	113.82	15.90	104.63–123.00	113.96	34.35	82.20–145.73	131.07	13.19	125.62–136.52	154.93	13.75	147.86–162.00	166.72	21.64	155.19–178.25
38	Total	109.65	15.72	103.43–115.86	109.03	25.26	97.21–120.85	134.33	15.96	129.53–139.12	154.02	15.83	149.08–158.95	164.98	21.03	157.27–172.70
38	p-value	1.0000			–			1.0000			1.0000			1.0000		

Tooth	Sex	RI			R1/4			R1/2			R3/4			RC			AC		
		A	SD	CI	A	SD	CI	A	SD	CI	A	SD	CI	A	SD	CI	A	SD	CI
21	F	54.60	10.17	51.16–58.04	70.23	10.39	66.12–74.34	83.71	8.61	81.18–86.23	93.94	17.34	84.70–103.18	108.37	9.16	102.22–114.52	212.45	57.87	206.42–218.48
21	M	58.49	9.77	55.03–61.96	74.81	8.86	71.67–77.95	86.01	12.62	82.18–89.85	95.94	13.25	90.35–101.54	141.07	58.04	80.17–201.98	214.27	56.53	208.29–220.24
21	Total	56.46	10.10	54.04–58.89	72.75	9.77	70.23–75.27	84.82	10.74	82.59–87.06	95.14	14.84	90.39–99.89	119.91	36.94	100.92–138.90	213.35	57.18	209.11–217.58
21	p-value	1.0000			1.0000			1.0000			1.0000			–			1.0000		
22	F	60.95	11.85	57.21–64.69	80.53	10.34	77.35–83.72	86.07	11.28	81.51–90.62	104.32	13.46	96.55–112.09	107.69	10.15	100.87–114.51	217.95	54.24	212.16–223.75
22	M	67.00	11.35	63.47–70.54	83.37	12.01	79.80–86.93	96.43	14.32	90.24–102.62	107.52	10.74	100.70–114.34	113.99	14.41	105.67–122.31	219.69	52.69	213.99–225.38
22	Total	64.01	11.92	61.41–66.62	82.00	11.26	79.63–84.37	90.93	13.69	87.00–94.86	105.80	12.15	100.89–110.70	111.22	12.87	105.91–116.53	218.81	53.44	214.76–222.86
22	p-value	1.0000			1.0000			0.7536			1.0000			1.0000			1.0000		
23	F	68.17	9.23	64.84–71.50	86.05	13.98	82.41–89.70	100.54	11.71	94.52–106.56	112.52	12.85	107.72–117.31	129.98	20.31	118.25–141.71	226.22	48.51	220.80–231.64
23	M	76.78	10.29	73.49–80.07	91.52	13.72	87.81–95.22	109.48	14.79	102.75–116.21	124.14	11.21	119.79–128.49	146.98	10.45	140.67–153.29	229.81	46.21	224.51–235.10
23	Total	72.95	10.67	70.44–75.46	88.69	14.06	86.08–91.30	105.48	14.06	100.86–110.10	118.13	13.34	114.62–121.63	138.17	18.21	130.96–145.37	227.97	47.40	224.18–231.75
23	p-value	0.0399			1.0000			1.0000			0.0554			1.0000			1.0000		
24	F	94.19	15.32	87.87–100.52	106.70	12.50	102.19–111.20	113.20	11.45	107.32–119.09	129.76	8.36	119.38–140.14	153.77	20.00	128.94–178.60	232.08	46.06	226.49–237.68
24	M	98.91	14.31	92.21–105.60	115.57	13.82	110.10–121.03	120.16	9.22	110.48–129.83	131.02	5.85	126.52–135.51	143.41	5.65	137.48–149.34	231.39	45.81	225.91–236.87
24	Total	96.29	14.90	91.81–100.77	110.76	13.74	107.18–114.34	115.02	11.15	110.19–119.84	130.57	6.55	126.78–134.35	148.12	14.33	138.50–157.74	231.73	45.89	227.83–235.63
24	p-value	1.0000			1.0000			–			–			–			1.0000		
25	F	94.57	8.58	90.16–98.98	111.05	11.90	106.02–116.08	106.21	25.32	91.59–120.82	126.59	11.61	117.67–135.51	154.67	15.00	142.13–167.21	230.49	44.79	225.15–235.84
25	M	105.88	11.97	98.97–112.79	115.90	12.84	110.35–121.45	124.08	8.11	115.57–132.59	132.69	7.72	127.51–137.88	146.27	5.70	139.20–153.34	231.61	45.55	226.21–237.01
25	Total	99.68	11.58	95.43–103.92	113.42	12.48	109.76–117.09	111.57	22.94	100.83–122.30	129.95	9.89	125.32–134.57	151.44	12.66	143.79–159.09	231.06	45.14	227.27–234.85
25	p-value	0.6680			1.0000			–			–			–			1.0000		
26	F	54.45	5.83	50.28–58.62	62.07	9.45	58.08–66.06	75.25	12.35	71.40–79.10	95.53	14.83	91.70–99.36	118.17	20.67	111.37–124.96	224.42	48.99	218.92–229.93
26	M	57.60	17.25	39.50–75.70	65.66	8.30	62.38–68.95	78.72	15.56	74.16–83.29	98.72	15.65	94.96–102.48	125.38	17.85	118.32–132.44	224.81	48.94	219.27–230.35
26	Total	55.63	11.05	49.74–61.52	63.97	8.96	61.45–66.49	77.08	14.17	74.10–80.07	97.23	15.30	94.57–99.90	121.16	19.73	116.28–126.05	224.62	48.93	220.72–228.51
26	p-value	–			1.0000			1.0000			1.0000			1.0000			1.0000		
27	F	100.28	12.52	96.17–104.40	115.60	12.03	109.19–122.01	119.52	23.27	104.73–134.30	138.63	10.78	130.35–146.92	159.98	17.84	153.94–166.02	235.75	42.74	230.66–240.84
27	M	105.59	12.07	101.88–109.31	122.51	12.62	116.02–129.00	128.79	5.42	123.78–133.81	139.71	8.04	133.53–145.88	157.69	15.27	151.24–164.14	233.82	43.29	228.71–238.93
27	Total	103.10	12.49	100.34–105.87	119.16	12.64	114.68–123.64	122.93	19.02	113.77–132.10	139.17	9.24	134.57–143.76	159.06	16.77	154.73–163.39	234.78	42.99	231.18–238.38
27	p-value	1.0000			1.0000			–			–			1.0000			1.0000		
28	F	169.11	18.27	161.73–176.49	193.63	22.13	183.82–203.45	199.04	31.09	180.26–217.83	207.58	24.06	193.69–221.47	221.63	23.59	212.30–230.96	262.47	28.24	257.25–267.68
28	M	174.04	23.08	164.52–183.57	175.84	9.31	170.46–181.22	186.44	20.69	174.98–197.90	196.25	17.37	188.12–204.38	207.19	24.45	195.41–218.98	258.04	28.03	253.19–262.88
28	Total	171.53	20.70	165.70–177.35	186.71	20.09	179.92–193.51	192.29	26.32	182.09–202.50	200.91	20.83	193.65–208.18	215.67	24.74	208.32–223.02	260.11	28.16	256.57–263.64
28	p-value	1.0000			0.2200			1.0000			1.0000			1.0000			1.0000		
31	F	43.68	11.07	34.42–52.94	59.19	8.85	56.05–62.33	74.01	11.33	70.06–77.96	86.50	13.33	81.43–91.56	94.55	13.93	72.39–116.72	205.55	63.37	199.13–211.98
31	M	52.91	9.88	46.63–59.19	64.28	10.06	60.65–67.91	79.59	11.04	76.64–82.55	84.42	13.77	77.57–91.27	105.01	18.11	86.01–124.02	209.11	60.13	202.91–215.32
31	Total	49.22	11.10	44.02–54.41	61.69	9.74	59.28–64.11	77.48	11.42	75.09–79.87	85.70	13.39	81.77–89.63	100.83	16.61	88.94–112.71	207.30	61.78	202.84–211.77
31	p-value	–			1.0000			1.0000			1.0000			–			1.0000		
32	F	54.98	8.55	50.43–59.54	66.78	11.16	63.30–70.26	81.33	8.83	78.30–84.37	93.67	18.72	80.28–107.07	94.43	14.79	86.83–102.04	211.06	59.60	204.87–217.25
32	M	60.63	8.35	56.48–64.78	72.28	12.54	68.64–75.92	85.04	13.86	80.54–89.53	92.95	6.79	86.67–99.23	123.64	39.71	90.45–156.84	210.86	59.44	204.69–217.03
32	Total	57.97	8.79	54.91–61.04	69.71	12.16	67.17–72.26	83.28	11.82	80.54–86.02	93.38	14.65	85.84–100.91	103.78	28.27	92.11–115.45	210.96	59.48	206.60–215.32
32	p-value	1.0000			1.0000			1.0000			–			–			1.0000		
33	F	61.77	8.84	58.77–64.76	82.19	10.94	79.44–84.95	98.25	14.25	91.58–104.92	110.83	13.31	105.67–115.99	123.93	16.80	113.78–134.08	224.29	49.91	218.76–229.83
33	M	71.99	12.62	68.58–75.40	90.02	13.76	86.40–93.63	108.58	13.73	102.91–114.24	124.68	14.84	118.69–130.68	140.05	14.50	131.29–148.81	229.77	46.02	224.52–235.02
33	Total	67.95	12.30	65.38–70.51	85.94	12.93	83.61–88.27	103.99	14.74	99.56–108.42	117.50	15.59	113.24–121.75	131.99	17.43	124.95–139.03	226.95	48.10	223.14–230.77
33	p-value	0.0018			0.0837			1.0000			0.0745			1.0000			1.0000		
34	F	81.70	11.55	78.19–85.21	96.43	13.37	92.15–100.70	108.12	10.04	103.55–112.69	125.14	13.31	118.91–131.37	149.17	28.19	127.50–170.84	227.65	47.64	222.27–233.03
34	M	87.58	12.41	83.97–91.18	104.84	14.41	100.23–109.45	121.44	15.16	112.68–130.19	128.87	11.24	120.83–136.91	149.48	20.06	135.13–163.82	227.69	47.40	222.32–233.05
34	Total	84.77	12.30	82.22–87.31	100.63	14.44	97.42–103.85	113.45	13.82	108.70–118.19	126.38	12.59	121.68–131.09	149.33	23.54	137.98–160.68	227.67	47.48	223.88–231.46
34	p-value	1.0000			0.7851			0.8249			1.0000			–			1.0000		
35	F	88.67	9.07	85.28–92.06	106.60	12.92	102.16–111.04	116.84	13.95	106.86–126.82	131.61	14.88	124.21–139.01	148.49	13.87	142.00–154.98	231.80	44.68	226.61–236.99
35	M	97.54	12.14	93.08–101.99	110.98	15.60	104.81–117.15	125.65	12.31	115.36–135.94	133.67	9.67	127.53–139.82	151.66	14.48	144.21–159.11	231.72	44.81	226.53–236.90
35	Total	93.18	11.55	90.22–96.13	108.51	14.20	104.90–112.11	120.75	13.63	113.98–127.53	132.43	12.90	127.62–137.25	149.95	14.05	145.26–154.63	231.76	44.71	228.10–235.42
35	p-value	0.2011			1.0000			–			1.0000			1.0000			1.0000		
36	F	45.32	3.79	11.30–79.34	56.39	7.54	53.47–59.32	67.11	5.22	64.86–69.37	83.28	12.74	79.66–86.90	97.36	12.17	93.05–101.68	212.02	57.95	205.98–218.06
36	M	51.95	7.55	39.94–63.96	60.12	9.59	55.75–64.49	70.00	8.65	67.02–72.97	86.27	13.07	82.83–89.70	100.80	10.54	96.72–104.89	214.85	55.51	208.94–220.75
36	Total	49.74	6.98	42.41–57.07	57.99	8.59	55.52–60.46	68.85	7.56	66.87–70.84	84.88	12.94	82.42–87.35	98.94	11.49	96.00–101.88	213.40	56.75	209.19–217.62
36	p-value	–			1.0000			1.0000			1.0000			1.0000			1.0000		
37	F	99.25	10.81	94.78–103.71	111.96	11.62	107.62–116.30	130.75	19.01	107.14–154.36	140.08	12.01	134.10–146.05	163.11	18.18	156.86–169.35	237.48	41.82	232.43–242.52
37	M	98.00	8.17	92.51–103.49	118.46	13.00	113.61–123.32	117.43	29.15	81.24–153.62	142.74	15.52	133.78–151.70	162.56	16.73	157.06–168.06	237.49	42.16	232.38–242.60
37	Total	98.87	9.98	95.49–102.24	115.21	12.66	111.94–118.48	124.09	24.24	106.75–141.43	141.24	13.49	136.38–146.11	162.83	17.32	158.78–166.87	237.48	41.95	233.90–241.06
37	p-value	1.0000			1.0000			–			1.0000			1.0000			1.0000		
38	F	175.95	20.27	164.72–187.17	186.58	19.87	175.11–198.05	204.87	27.12	193.42–216.32	204.74	18.40	193.61–215.86	229.10	26.26	220.35–237.86	261.88	28.98	256.46–267.31
38	M	171.02	19.00	161.24–180.79	178.58	27.68	161.85–195.31	190.95	15.75	184.30–197.60	194.63	18.27	185.54–203.71	217.29	17.71	209.44–225.14	260.68	28.20	256.00–265.35
38	Total	173.33	19.45	166.32–180.34	182.73	23.82	173.30–192.15	197.91	23.04	191.22–204.60	198.87	18.72	192.00–205.73	224.70	23.97	218.45–230.95	261.21	28.50	257.69–264.73
38	p-value	1.0000			1.0000			1.0000			1.0000			1.0000			1.0000		

*A* average; *SD* standard deviation; *F* female; *M* male; p-values calculated in cases with at least n = 10 for each sex. *CI* 95% confidence interval; Confidence intervals calculated in cases with at least n = 10 for each sex

The data show that was only a significant difference between the sexes for age in stage RI for teeth 23 and 33(p < 0.05) and average age was higher in men than in women. The R3/4 stage of teeth 23 and 33 had p-values very close to the threshold, and the mean age was also higher in men.

## Discussion

In living subjects, age estimations have been requested for cases of refugees, unaccompanied minors, and child trafficking, among others [2, 13]. In cases of subadults, age estimation based on analysis of dental mineralization stages is indicated [2]. However, the data available on dental mineralization stages go back a few decades and are specific to certain populations [7–13, 17, 25]. Several methods have been tested in different populations, and the use of specific tables is recommended [2, 5, 13, 21–24]. Several studies, such as by Fei et al. [28], Phillips and van Wyk Kotze [12] and Koshy and Tandon [5], indicate age overestimation or underestimation and low accuracy rates. Gelbrich et al. [29] recommend the simultaneous use of two methods to obtain more precise estimates. For the Brazilian population, the use of the N.M.M. table [17] has presented low success rates [16, 18, 19], as well as methods based on foreign populations [19, 20]. Authors have demonstrated the need to obtain current data on dental mineralization stages in Brazilian individuals [2, 19, 30].

In the present study we evaluated dental mineralization stages of modern Brazilian individuals (born between 1990 and 2018) using digital panoramic radiographs, which were coded, in order to avoid any bias on the part of the examiners by knowing the exact chronologic age, as done by Koshy and Tandon [5].

Concerning the measurement of reliability of qualitative variables, Ferrante and Cameriere [31] recommend the use of kappa coefficient. In our study, for both intra and inter-examiner precision analysis, kappa ranged from 0.92 to 1.00 (which corresponds to almost perfect agreement). With respect to the same analysis, Karkhanis et al. [13], for intra-examiner, obtained values that ranged from 0.81 to 0.93, and for inter-examiner, from 0.81 to 0.90. For intra-examiner reproducibility analysis, AlQahtani et al. [32] and Blenkin and Taylor [33] obtained kappa values of 0.81 and 0.80, respectively.

We adapted the Moorrees et al. [8] method, reducing the number of stages from 14 to 11, to simplify and to facilitate the process. The three stages that were included in other stages presented a very slight (almost imperceptible) difference in relation to the stage in which they were included. Some authors have already done the same. Haavikko [15] used 12 of the 15 stages proposed by Gleiser and Hunt [14]. Other authors, as Nolla [7] and Moorrees et al. [8], modified the stages proposed by Gleiser and Hunt [14]. Nicodemo, Morais and Médici Filho [17] used 8 mineralization stages, based on the 10 stages proposed by Nolla [7]. AlQahtani et al. [32] modified Moorrees et al. [8] stages.

We used a convenience sample, with 50% males and 50% females. Our initial sample consisted of 1100 digital panoramic radiographs. Nevertheless, our sample is considerably larger than those used by several other authors.

Nolla [7], in her study of dental mineralization, evaluated 50 sets of radiographs obtained from the University of Michigan School, US. Gleiser and Hunt [14] employed a sample of 50 children (25 boys and 25 girls). They evaluated the mineralization of first molars and proposed 15 stages of mineralization. Moorrees et al. [8] proposed a method for age estimation based on their study which evaluated 99 intraoral radiographs of Boston children and 246 lateral jaw radiographs of boys and girls. However, not all radiographs were used to assess the mineralization stage of all teeth, because the images of some teeth could not be clearly identified.

Niquini et al. [19] performed a study to determine the accuracy of the chronological table of mineralization of permanent teeth among Brazilians. The authors used 442 panoramic radiographs of individuals aged between 5 and 30 years and 4 months taken in the downtown area of the city of Belo Horizonte-MG, Brazil, applying the Brazilian N.M.M. table. The total mean percentage of correct answers was 63.5%, which is a low number, especially in the age group from 14 to 17 years.

Liversidge [34] conducted a study in London with a sample of 1050 panoramic radiographs of white and Bengali subjects aged between 2 and 22 years and analyzed mineralization stages only of mandibular permanent teeth. Phillips and van Wyk Kotze [12], feeling the need for specific data on South African subjects, built a current table of dental mineralization stages for the mentioned population, using the stages proposed by Moorrees et al. [8], with a sample of 1006 panoramic radiographs of subadults aged 7 to 16 years. Karkhanis et al. [13] did the same for the Australian population, with 392 panoramic radiographs of individuals between 4 and 25 years old. Šešelj et al. [25] proposed a new chronology of tooth development only for canines, premolars, and permanent molars, with a sample of radiographs taken between 1940 and 1982 from 590 European individuals up to 28 years old.

AlQahtani et al. [32] performed a study to develop a comprehensive evidence-based atlas to estimate age using both tooth development and alveolar eruption. The authors used a sample of 704 archived records: radiographs of known age individuals and known age-at-death skeletal remains. Maled and Vishwanath [3] developed a study to determine the chronology of third molar mineralization to establish reference data for Indian population. The authors evaluated 167 digital panoramic radiographs and used the 8-stage developmental scheme proposed by Demirjian et al. [9].

Nicodemo, Moraes and Médici Filho [17] created a table for the chronology of permanent teeth mineralization of Brazilian population. They evaluated a sample of 478 Brazilian individuals. Their study “Table of the chronological mineralization of permanent teeth among Brazilians” is the sole reference of dental mineralization of Brazilian individuals published so far. The authors conducted their research in the late 60's and early 70’s; the obtained data are not current data; they are more than 50 years old.

This study analyzed 1004 panoramic radiographs of Brazilian individuals aged between 2 and 25 years (35 and 307 months), born between 1990 and 2018, and created tables of mineralization stages of permanent teeth. It is worth noting that the data obtained refer to the modern and current population.

The present study was performed with images of digital panoramic radiographs of children aged 35 months or older, that is, subjects younger than those analyzed by Phillips and van Wyk Kotze [12] and Karkhanis et al. [13]. Nevertheless, further studies with younger individuals are required to cover the initial mineralization phases of incisors, canines, and first molars. The data presented by the aforementioned authors provide the same observation.

Studies on the chronology of dental mineralization stages without numerical data limit their applicability [11]. The findings of the present study are provided in tables with numerical data. This allows calculating age estimates without conversion indices, which facilitates the expert investigation.

Regarding the analysis of age distribution for each mineralization stage and according to the overall average of all permanent teeth, there were no significant differences between the sexes, except for canines, agreeing with the findings by Liversidge. [34] and de Šešelj et al. [25].

The data on the mineralization stages of permanent teeth found in this study were obtained from a sample of Brazilian subjects of both sexes, aged between 2 and 25 years, and born between 1990 and 2018. The findings were presented numerically, which facilitates their use in expert age estimation investigations without conversion indices. However, studies with a younger population are required to verify the initial mineralization stages of anterior teeth and molars.

Recent studies have questioned the classification of ancestry into three categories: European, African and Asian [35, 36], and the miscegenation of populations is an important factor in this context. Brazil is a diverse country that received many immigrants from different parts of the world, as well as slaves from Africa, who mixed with its native population (indigenous people). The country does not have clear demarcation lines between populations in terms of ethnic, linguistic, cultural or historical characteristics [37], mainly due to the great miscegenation of its population. As the present study was carried out with a sample composed of individuals from the southeastern region of Brazil, we believe that new studies are indicated, with samples from other Brazilian regions.

The tables presented can be used to estimate the age of Brazilians in investigations with living and dead subjects, helping justice and society.

## Conclusions

In the present study, we evaluated the mineralization stages of permanent teeth of Brazilian subjects from digital panoramic radiographs and found no correlation between the chronology of mineralization stages and sex, except for upper and lower canines, which presented higher average ages for men. From the obtained results, numerical tables of the chronology of dental mineralization stages were prepared.

## Data Availability

The datasets generated and analyzed during the current study are available from the corresponding author on reasonable request.
